# Postoperative Recurrences in Patients Operated for Pheochromocytomas and Paragangliomas: New Data Supporting Lifelong Surveillance

**DOI:** 10.3390/cancers14122942

**Published:** 2022-06-14

**Authors:** Stefanie Parisien-La Salle, Jessica Chbat, André Lacroix, Paul Perrotte, Pierre Karakiewicz, Issam Saliba, Xuan Kim Le, Harold J. Olney, Isabelle Bourdeau

**Affiliations:** 1 Division of Endocrinology, Department of Medicine and Research Center, Centre Hospitalier de l’Université de Montréal (CHUM), Montreal, QC H2X 0C1, Canada; stefanie.parisien-la.salle@umontreal.ca (S.P.-L.S.); jessica.chbat@mail.mcgill.ca (J.C.); andre.lacroix@umontreal.ca (A.L.); 2 Division of Urology, Centre hospitalier de l’Université de Montréal (CHUM), Montreal, QC H2X 0C1, Canada; paul.perrotte@umontreal.ca (P.P.); pierre.karakiewicz@umontreal.ca (P.K.); 3 Division of Otolaryngology-Head and Neck Surgery, Centre hospitalier de l’Université de Montréal (CHUM), Montreal, QC H2X 0C1, Canada; issam.saliba@umontreal.ca; 4 Division of Medical Oncology, Department of Medicine, Centre de recherché du CHUM (CRCHUM), Montreal, QC H2X 0C1, Canada; xuan-kim.le.chum@ssss.gouv.qc.ca (X.K.L.); harold.olney.med@ssss.gouv.qc.ca (H.J.O.)

**Keywords:** pheochromocytoma, paragangliomas, recurrences, follow-up

## Abstract

**Simple Summary:**

At least 10% of pheochromocytomas (PHEOs) and paragangliomas (PPGLs) may recur after the initial surgery. The optimal follow-up time for these tumors remains unknown. We present a cohort of recurrent PPGLs in a clinical care setting of a quaternary center. In this paper, we describe recurrence patterns based on tumor location (head and neck paragangliomas, thoracoabdominal paragangliomas, and pheochromocytomas). We report that the overall mean delay of recurrence was 9.7 years and that one-third of the cohort had a recurrence more than 10 years after the initial surgery. Additionally, 17.6% of recurrent PHEOs were smaller than the predicted cutoff for recurrence (5 cm). Finally, more than 50% of recurrent PPGLs harbored a germline mutation in a susceptibility gene. In sum, this paper supports that overall, the safest option remains a lifelong follow-up.

**Abstract:**

At least 10% of pheochromocytomas (PHEOs) and paragangliomas (PGLs) (PPGLs) may recur after the initial surgery. Guidelines recommend annual screening for recurrence in non-metastatic tumors for at least 10 years after the initial surgical resection and lifelong screening in high-risk patients. However, recent data suggest that a shorter follow-up might be appropriate. We performed a retrospective analysis on patients with PPGLs who had local and/or metastatic recurrences between 1995 and 2020 in our center. Data were available for 39 cases of recurrence (69.2% female) including 20 PHEOs (51.3%) and 19 PGLs (48.7%) (13 head and neck (HNPGL) and 6 thoracoabdominal (TAPGL)). The overall average delay of recurrence was 116.6 months (14–584 months) or 9.7 years and the median was 71 months or 5.9 years. One-third of the cohort had a recurrence more than 10 years after the initial surgery (10–48.7 years). The average tumor size at initial diagnosis was 8.2 cm for PHEOs, 2.7 cm for HNPGLs, and 9.6 cm for TAPGLs. Interestingly, 17.6% of PHEOs were under 5 cm at the initial diagnosis. Metastatic recurrence was identified in 75% of PHEOs, 15.4% of HNPGLs, and 66.7% of TAPGLs. Finally, 12/23 (52.2%) patients with recurrence who underwent genetic testing carried a germline mutation. Overall, the safest option remains a lifelong follow-up.

## 1. Introduction

Pheochromocytomas (PHEOs) and paragangliomas (PGLs) (PPGLs) are rare catecholamine-secreting tumors that arise from the adrenal medulla or extra-adrenal chromaffin cells [1]. PPGLs can be metastatic, which is defined by the presence of metastases in nonchromaffin tissue [2]. The prevalence of metastatic disease in PPGLs varies between 10 and 17% but can reach 35–75% in patients carrying disease-causing germline mutations in *SDHB* [2,3,4,5,6]. In a recent study from the UK, the prevalence of metastatic disease in patients with *SDHC*-related PPGLs was 19.6% [7]. Patients with metastases have shorter survival than their benign counterparts [8]. The 5-year survival rate for metastatic PPGLs is estimated to be around 40–77% [9]. However, there are no good histopathological criteria to predict the potential malignancy or tumoral recurrences of PPGLs [9]. Larger tumor size, extra-adrenal tumors, higher 3-methoxytyramine levels, younger age at diagnosis, and germline mutations, notably in the *SDHB* gene, are predictors of an increased risk of malignancy [10,11]. In a study by Schovanek et al., younger age at diagnosis was a predictor of patient survival but not for the development of metastases [12]. Nevertheless, patients who undergo complete resection of their PPGLs can still be at risk for metastases, and recurrences and should be followed-up after surgery [13]. Predictors of recurrence are younger age, familial disease, and tumor site and size [13]. The recurrence rate for PPGLs is approximately 6.5–17.4%, and early detection is important to reduce morbidity and mortality caused by mass effect, catecholamine secretion, and metastatic recurrence [9,14,15].

The 2014 Endocrine Society Guidelines suggest lifelong follow-up to assess for recurrence or metastatic disease [2]. More recently, the European Society of Endocrinology Guidelines suggests a biochemical follow-up of at least ten years for low-risk patients and lifelong follow-up for high-risk patients (young patients, large tumors, genetic mutation, and PGL) with added imaging in patients with a biochemically silent tumor or a known genetic mutation [16].

Moreover, PPGLs are the most highly heritable tumors in adults. A germline mutation in a known susceptibility gene can be found in approximately 30–35% of Caucasians with PPGLs [6]. Genetic counseling is recommended for all patients with PPGLs as genetic status can have an impact on patient care [2,6].

In 2016, a systematic review and meta-analysis of PHEOs and thoracoabdominal PGLs (TAPGLs) showed a lower incidence of recurrence (5% per 5-year follow-up) [17]. Recurrences were more frequent in patients with PGLs and familial disease [17]. A recent meta-analysis by Holscher et al. showed a 3% recurrence rate in sporadic PHEOs after curative adrenalectomy with a median recurrence time of 49.4 months and a follow-up time of 77.3 months [18]. These two recent meta-analyses show lower recurrence rates than what was initially believed, calling into question the long-term follow-up for PPGLs.

Interestingly, a 2021 review by Nölting et al. proposes a personalized follow-up depending on disease characteristics and mutational status of patients [6]. They give general guidance to patients deserving lifelong follow-up. These include patients with germline mutation, history of PGL, age < 20 years at initial diagnosis, tumor size ≥5 cm, multiple or recurrent PPGLs, or noradrenergic/dopaminergic phenotype [6]. These characteristics are often found in patients with cluster 1A *SDHx*-related PPGLs. Patients with cluster 2 related PPGLs (*RET (high/moderate risk for PHEO)*, *NF1*, *TMEM127*, and *MAX* mutation carriers with a history of a PHEO) should be followed up with yearly clinical and biochemical evaluation and abdominal/pelvic MRI once every 5 years. Finally, it is uncertain how patients with cluster 3-related PPGLs should be followed. However, these tumors do present with a high risk of recurrence, and it is hypothesized they should be followed like patients with cluster 1A *SDHx*-related PPGLs [6].

In sum, the optimal timing, duration, and modality of follow-up vary from study to study due to a lack of systematic studies and the rarity of the disease [6,9,18]. Our primary objective for this study was to retrospectively evaluate the rate and delay of recurrence in our cohort of patients with PPGLs in a real-life clinical setting.

## 2. Materials and Methods

### 2.1. Study Population

In this retrospective single-center study, we reviewed the records of 309 patients with a histological diagnosis of PPGLs treated at the Centre hospitalier de l’Université de Montréal (CHUM) from January 1995 to May 2020. We included patients with PPGLs who were 15 years or older, who underwent an initial resection of their tumor, and who were considered in remission postoperatively but had a recurrence of their PPGL during the follow-up period. We also included patients who already had metastatic disease at first diagnosis but were considered in remission following initial surgery. Exclusion criteria were missing data and patients who never went into remission. This resulted in a total of 39 cases of recurrent PPGLs. Of the 270 remaining patients, 197 were either followed at another hospital and only referred to us for genetic counseling, lost to follow-up, refused follow-up, had missing data, or never went into remission. Seventy-three patients were considered in remission and were followed regularly with imaging or biochemistry at our center and have not presented a recurrence yet.

Remission was described as absence of disease on imaging and a normal biochemical panel (urinary catecholamines and/or plasma metanephrines in normal range) for patients with functional PPGLs following surgery. Recurrence was defined as local or presence of metastasis in patients who were considered in remission after surgery. A functional tumor was defined by urinary catecholamine or plasma metanephrine levels above the upper limit of normal (ULN). Dosing of catecholamines was performed using HPLC (high-performance liquid chromatography) with solid-phase extraction (SPE) and dosing of plasmatic metanephrines was performed using LC-MS/MS (liquid chromatography–mass spectrometry) with SPE. Patients were followed with yearly or bi-yearly biochemical testing and or imaging depending on functionality, genetic status, and other risk factors. Genetic counseling was offered to a subgroup of patients beginning in 2005. From 2005 to 2015, genetic analysis included sequential gene testing and multiplex ligation-dependent probe amplification (MLPA) of specific genes (Molecular Diagnosis Laboratory of Alberta Health Services, Calgary and Hôpital européen Georges Pompidou Centre Georges Pompidou, Paris). From 2015 to 2020, a multigene panel was performed, including at least 14 susceptibility genes: *SDHA*, *SDHAF2*, *SDHB*, *SDHC*, *SDHD*, *RET*, *VHL*, *FH*, *NF1*, *MAX*, *TMEM127*, *EGLN1*, *KIF1B*, and *MEN1* genes (Invitae San Francisco); or *SDHA*, *SDHAF2*, *SDHB*, *SDHC*, *SDHD*, *RET*, *VHL*, *FH*, *NF1*, *MAX*, *TMEM127*, *EGLN1*, *ENGLN2*, *EPAS1*, and *MDH2* genes (Hôpital européen Georges Pompidou Centre Georges Pompidou, Paris). Gene sequencing and deletion/duplication analysis were performed using next-generation sequencing (NGS) technology for all genes except for the *SDHA* gene (Invitae San Francisco), which was not evaluated for deletion/duplication. Genetic counseling was performed by a genetic counselor, with written consent from the patient before genetic analysis. Seeing genetic counseling/testing was recommended for all PPGLs starting in 2014, some patients followed prior to this date did not undergo genetic testing [2]. We collected data on initial clinical presentation, imaging, secretion pattern, postoperative surveillance, genetic analysis, and delay and management of recurrence. The ethical committee of our institution approved the study.

### 2.2. Statistical Analysis

Categorical variables are reported as percentages. Continuous variables are reported as mean, standard deviation, and minimum and maximums. Continuous variables were compared using Mann–Whitney test or ANOVA. All calculations were carried out with SPSS software (SPSS:v25, IBM, Armonk, NY, USA).

## 3. Results

### 3.1. Patient Cohort

**Figure 1:**
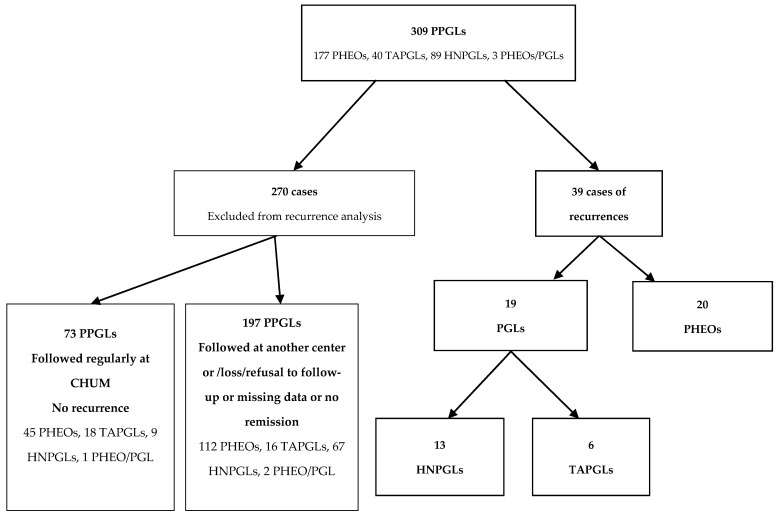
Flowchart representing the cohort of patients with PPGLs studied.

**Figure 2:**
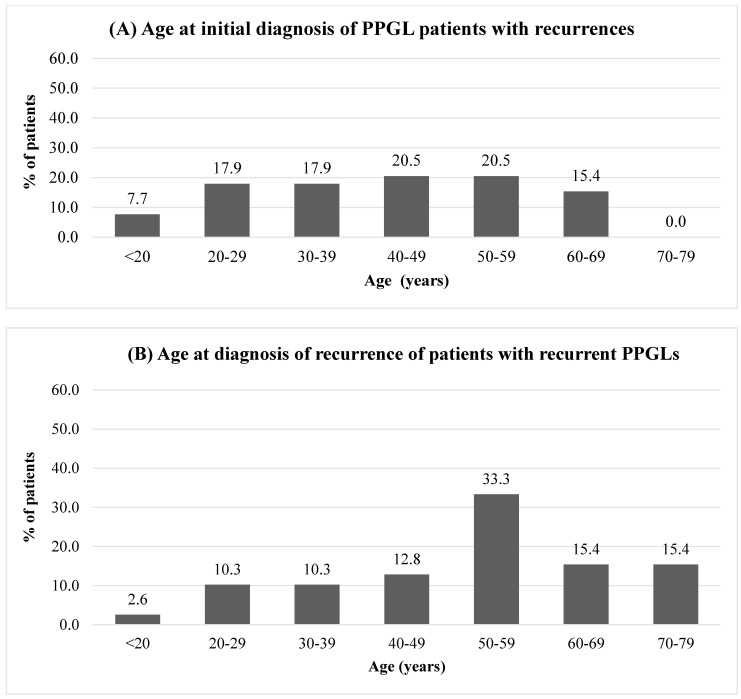
Age of patients with PPGLs at initial diagnosis and recurrence. Notes: PPGLs: pheochromocytomas and paragangliomas. (**A**) Age at initial diagnosis of PPGL patients with recurrences. (**B**) Age at diagnosis of recurrence of patients with recurrent PPGLs.

Our study included a cohort of 309 patients with PPGLs: 177 patients (57.3%) with PHEO, 129 (41.7%) with PGL, and 3 (1.0%) with both PHEO and PGL at diagnosis. We identified 39 patients with recurrences: 20 PHEOs and 19 PGLs. Of the 19 PGLs, 13 were head and neck PGLs (HNPGLs), and 6 were TAPGLs (Figure 1). Thus, the recurrent rate for PHEOs was 20/177 (11.3%), 13/89 (14.6%) for HNPGLs, and 6/40 (15%) for TAPGLs, with an overall rate of 12.6%. Twenty-seven patients (69.2%) were female. The average age at initial diagnosis was 41.8 (15–67) years old. The average age at recurrence was 51.9 (19–78) years old. Approximately one-third of patients had a recurrence before the age of 50 and two-thirds after the age of 50 (Figure 2). Of the 39 patients, 18 (46.2%) were followed regularly with at least yearly biochemistry and/or imaging.

**Figure 3:**
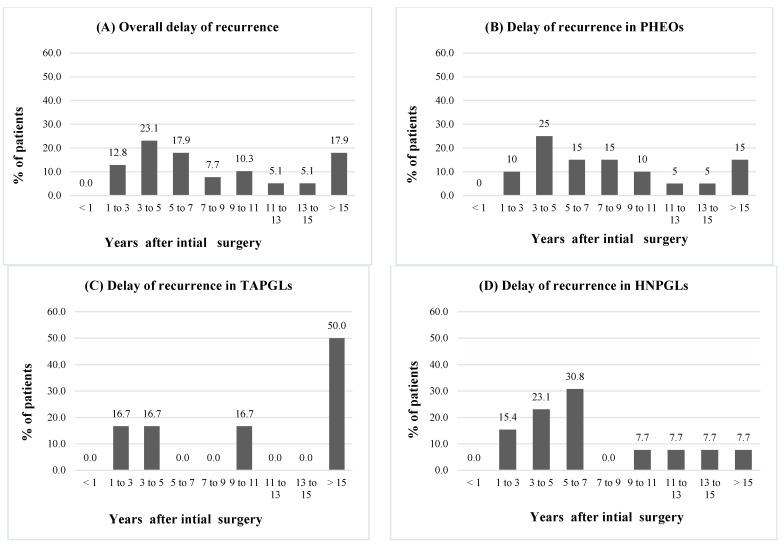
Time of recurrence in years after initial surgical resection. Notes: PPGLs: pheochromocytomas and paragangliomas, PHEOs: pheochromocytomas, TAPGLs: thoracoabdominal paragangliomas, HNPGLs: head and neck paragangliomas. (**A**) Overall delay of recurrence. (**B**) Delay of recurrence in PHEOs. (**C**) Delay of recurrence in TAPGLs. (**D**) Delay of recurrence in HNPGLs.

The overall mean delay of recurrence was 116.6 months (14–584 months) or 9.7 years and the median was 71 months or 5.9 years. Fourteen patients (35.9%) had a recurrence within 5 years of follow-up, whereas 25 patients (64.1%) had a recurrence after 5 years of follow-up, including 13 patients (33.3%) that had a recurrence after 10 years of follow-up. For seven patients (17.9%), recurrence was diagnosed more than 15 years later. One patient experienced a recurrence 48.6 years after the initial diagnosis (Figure 3). No statistical differences were observed when comparing recurrence delays between PHEOs, HNPGLs, and TAPGLs (*p =* 0.23). Eighteen patients (46.2%) had local recurrences and 21 patients (53.8%) had metastatic recurrences.

Twenty-three patients (23/39 (59%)) with recurrent disease underwent genetic testing. These revealed that 10 had no identified mutations (10/23 (43.5%)), 12 had an identified mutation (12/23 (52.2%)) and one is still pending. Patients without an identified mutation had an average delay of recurrence of 152.5 months (12.7 years) compared to 107.3 months (8.9 years) in patients with a mutation (*p =* 0.62).

Of the 39 patients with recurrent PPGLs, 36 were considered as having sporadic PPGLs (no family history of PPGLs or syndromic presentations). Two patients presented with familial cases of MEN2A disease and one patient had missing information regarding familial history. Patients with sporadic PPGLs had a mean delay of recurrence of 118 months (9.8 years). The two patients with familial MEN2A-associated PPGLs had recurrence delays of 14 months and 240 months.

Regarding the other 270 patients in our cohort, 197 were either followed at another hospital and only referred to us for genetic counseling, lost to follow-up, refused follow-up, had missing data, or never went into remission. Seventy-three patients were considered in remission and were followed regularly with imaging or biochemistry at our center. Of these patients, 66 underwent genetic testing and 21/66 (31.8%) presented a pathogenic variant in a susceptibility gene (5 *NF1*, 4 *RET*, 4 *SDHB*, 3 *SDHC*, 2 *MAX*, 1 *FH*, 1 *SDHA*, 1 *VHL*). Delays to recurrence and follow-up times are summarized in Table 1.

### 3.2. Characteristics of PHEOs with Recurrences 

**Figure 4:**
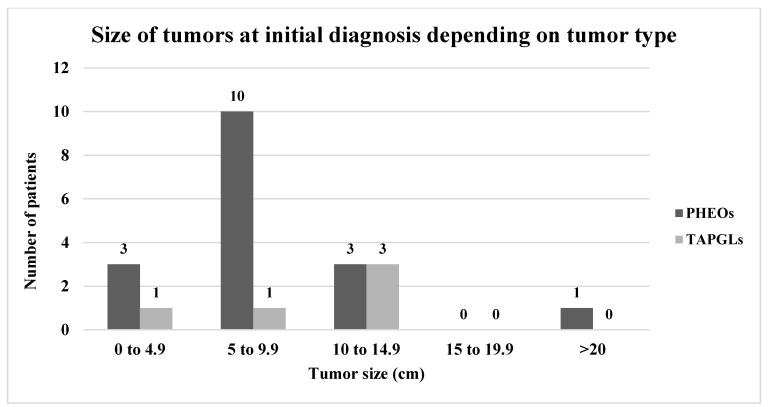
Size of tumors at initial diagnosis depending on tumor type. Notes: PPGLs: pheochromocytomas and paragangliomas, PHEOs: pheochromocytomas, TAPGLs: thoracoabdominal paragangliomas. * Tumor size missing for 3 PHEOs. ** Tumor size missing for 1 TAPGL.

The average age at initial diagnosis of 20 patients with recurrent PHEOs was 42.4 (16–67) years old, and 70% of patients were women. Initial tumor size was known for 17 patients. The average initial tumor size at diagnosis was 8.2 cm (3–24 cm). Interestingly, three patients (17.6%) had tumors that were under 5 cm (3–3.5 cm), and four patients (23.5%) had a tumor size that was 10 cm or more (10–24 cm) (Figure 4). Of the three patients with tumors under 5 cm, two did not undergo genetic testing and one had a negative multigene panel result (Table 2).

In regard to tumor location, seven PHEOs were left-sided, ten were right-sided and three were bilateral. Fifteen PHEOs were functional and five patients had an unknown functionality status at initial diagnosis. At recurrence, 18/20 (90%) were functional (2 adrenergic, 10 noradrenergic, 6 adrenergic and noradrenergic). Two PHEOs that were originally functional did not secrete catecholamines at recurrence. The average age at recurrence was 53.3 (19–78) years old, with an average delay of recurrence of 120.9 (14–584) months or 10 years with a median of 77.5 months (6.4 years) (Table 2).

Of the 20 patients with recurrent PHEOs, 5 patients (25.0%) had local recurrence and 15 (75.0%) had a metastatic recurrence. Of these recurrences, 16 (88.9%) were identified by biochemical testing either in the context of symptoms or yearly testing then confirmed by imaging, and 2 (11.1%) were identified by imaging with negative catecholamines. Mode of recurrence was not available for two patients (missing data on how the recurrence was found). Ninety percent of patients had functional tumors at recurrence. Of the two patients whose recurrence was identified by imaging, one was a 20-year-old female who was diagnosed with a left functional adrenal PHEO and an abdominal mass that was later identified as a PGL in pathology. She underwent resection for both. This patient was closely monitored over the years; her biochemistry remained normal, but imaging showed a small nodule described as an adrenal adenoma on her right adrenal gland. ^18^F-FDG PET/CT scan revealed a significant right adrenal uptake (SUVMax 5) and a discreet uptake of a 6 mm nodule in the left paraaortic area. Initially, catecholamines remained normal but chromogranin A was increased (89.2 ng/mL, N < 82). The patient underwent a right adrenalectomy and resection of the paraaortic nodule, which confirmed the PPGL recurrence. Her genetic panel revealed a germline pathogenic *FH* mutation [19].

The other patient whose recurrence was diagnosed with imaging was a 64-year-old man with a 24 cm left functional malignant PHEO with hepatic metastases [20]. His genetic panel was negative. He underwent adrenalectomy and a partial hepatectomy and was considered in remission after surgery, with normalization of catecholamines and imaging. He was followed with urinary catecholamines, plasmatic metanephrines, and chromogranin A levels every 3 months and with ^18^F-FDG PET/CT and abdominal CT-Scan alternating every 6 months. Four years following surgery, an abdominal MRI was ordered following an anomaly described in the patient’s liver on the CT-scan and showed small hepatic lesions. These lesions did not have a significant uptake on a follow-up ^18^F-FDG PET/CT but showed a slight uptake on MIBG. Catecholamines remained in the normal range; however, chromogranin A was elevated at 252.2 ng/mL (N < 82). The patient underwent a partial hepatectomy and the pathology revealed PHEO metastases. This case demonstrates a patient with initial metastatic disease who was in remission following surgery but presented 55 months later with a metastatic recurrence.

Genetic analysis of 11 patients with PHEOs showed no mutations in 7/11 (63.3%) individuals. However, three patients carried a germline *RET* mutation (two bilateral PHEOs, one left PHEO), and one *FH* mutation (left PHEO). The *RET* mutations were identified by sequential genetic testing and the other eight had multigene panels.

### 3.3. Characteristics of HNPGLs with Recurrences 

Among the 19 patients with PGL recurrences, 13 had HNPGLs (68.4%). For these patients, the average age at initial diagnosis was 46.2 (27–57) years old and 84.6% were women. Among the 12 patients with known initial tumor size, the average size at diagnosis was 2.7 cm (0.2–7.5 cm). The average age at recurrence was 53 (30–71) years old, with an average delay of recurrence of 82.4 (24–180) months and a median of 67 months (5.5 years) (Table 2).

Eleven patients (84.6%) had local recurrences and two (15.4%) had metastatic recurrences. Of these recurrences, ten (76.9%) were identified by imaging, two (15.4%) by biochemical testing, and one was unknown. For the two recurrences identified by biochemistry, one had elevated Chromogranin A and urinary normetanephrines and one had elevated urinary dopamine (Table 2).

Six patients with HNPGLs underwent genetic testing with a multigene panel. Two patients (33.3%) were negative, two had an *SDHC* mutation (33.3%), one patient had an *SDHA* (16.7%) mutation and one patient had an *SDHD* mutation (16.7%).

### 3.4. Characteristics of TAPGLs with Recurrences 

Among the 19 patients with PGL recurrences, 6 had TAPGLs (31.6%). The average age at initial diagnosis was 30.3 (15–45) years old. Two patients (33.3%) were women.

Initial tumor size was known for five patients. The average initial tumor size at diagnosis was 9.6 cm (3–14 cm). One patient had an initial tumor size of less than 5 cm (3 cm). This patient carried a germline *SDHB* mutation. Three patients (60.0%) had a tumor that was 10 cm or more (Figure 4). Functionality status at diagnosis was known for only three patients, all being functional. The average age at recurrence was 45.2 (27–60) years old, with an average delay of recurrence of 176 (26–312) months with a median of 188 months (15.7 years) (Table 2).

Two patients (33.3%) had local recurrences and four (66.7%) had metastatic recurrences. Of these recurrences, four (66.7%) were identified by imaging and two (33.3%) by biochemical testing (one patient had elevated urinary norepinephrine and normetanephrine and one had elevated plasmatic and urinary normetanephrines). Two-thirds of these tumors were functional at recurrence and one-third were non-functional (Table 2).

All six patients with TAPGLs underwent genetic testing with a multigene panel. One (16.7%) was negative. Three patients had an *SDHB* mutation (50%), one had an *SDHA* mutation (16.7%), and one had pending results but had a loss of *SDHB* protein expression in his resected PGL. The mean delay for recurrent *SDHB* TAPGLs was 143 months vs. 159 months for the other TAPGLs (one *SDHA* and one with no know mutation). Two-thirds of *SDHB* TAPGLs were metastatic at recurrence.

One patient had initial metastatic disease at diagnosis. He had a 13 cm functional TAPGL with positive lymph nodes. He carried a germline *SDHB* mutation. He underwent resection of his PGL and lymph nodes and was considered in remission of his tumor after surgery, with normal imaging and biochemistry. He was followed with urinary catecholamines and plasmatic metanephrines every 3 months and with ^18^F-FDG PET/CT and abdominal CT-Scan alternating every 6 months. He had a metastatic recurrence with bone lesions 26 months post-op with the initial recurrence marker being imaging.

## 4. Discussion

PPGLs are rare tumors with a high recurrence rate [2]. However, the optimal follow-up time and the most appropriate detection tools for recurrence remain uncertain. Moreover, recurrence rates are variable depending on reports, with rates ranging from 6–17% [15,21] (Table 3). In 2016, a meta-analysis of 38 studies performed by Amar et al. showed a median recurrence rate of 6%, ranging from 1 to 34% [17]. A 5-year cumulative incidence of recurrences of 4.7% was reported. This was lower than what was previously described and challenges the pertinence of long-term follow-up in PPGLs after initial complete resection [17] (Table 3).

We identified 39 patients with recurrences in our cohort of 309 patients with PPGLs, representing 11.3% for PHEOs, 14.6% for HNPGLs, and 15% for TAPGLs. However, we underline the fact that these numbers are from a cross-sectional study from our cohort of patients and do not reflect longitudinal follow-up since 197 patients of our cohort were mainly followed at other centers or lost to follow-up. Our recurrence rate was established similarly to the other cohorts stated in Table 3, who did not have a systematic long-term follow-up, with the exception of the study by Parasiliti-Caprino et al. who described a cohort of 242 PPGLs (220 PHEOs and 22 sympathetic PGLs) where all patients were followed up annually with hormonal values in case of secreting tumors and with CT/MRI if the neoplasm was biologically silent [15]. This team found a 17.4% (42/242) recurrence rate, which is concordant with our study as well as others in the literature. Younger age (36.4 vs. 51.9 years old), genetic mutations and tumor size (50.5 vs. 48 mm) were once again predictors for recurrence and lifelong follow-up was suggested based on their results [15]. Additionally, we included HNPGLs as well as PHEOs and TAPGLs in our study, which differs from most previously published cohorts (that include mostly PHEOs and functional TAPGLs or only HNPGLs). Each tumor group was studied independently. We followed 73 patients who were considered in remission regularly with at least yearly biochemistry and/or imaging. This is in contrast to previous studies in which standardized follow-up was lacking, as described in the meta-analysis by Amar et al.: “Patient follow-up was neither standardized nor exhaustive in the included studies [17].” Our study demonstrates a real-life clinical experience of a quaternary reference center for PPGLs. Thus, metastatic cases can be overrepresented. 

We identified 17 patients with sporadic PHEOs that had recurrences, representing 9.6% (17/177) of our cohort. This number was higher than what was described in the meta-analysis by Holscher et al., which reported a recurrence rate of 3% for sporadic PPGLs [18] with a mean time to recurrence of 49.4 months and a mean follow-up period of 77.3 months. Their follow-up time was considerably shorter than ours, as our mean recurrence time for sporadic PHEOs was 124.5 months (10.3 years) [18]. If we considered only the recurrences within the same time frame as the one used in this meta-analysis, we would have eight cases of recurrence, representing 4.5% of our cohort thus underlining the importance of a longer follow-up [18]. In our study, the definition of sporadic was based on the absence of family history and syndromic presentation as genetic testing was not universally offered to patients before 2014. Whereas, in the meta-analysis by Holscher et al., only one study was rated as representative of sporadic pheochromocytomas, since all the included patients routinely underwent genetic testing.

The majority of patients had a recurrence more than 5 years after initial resection (64.1%) and one-third of patients had a recurrence after 10 years of follow-up. This distribution was found in PHEOs, HNPGLs, and TAPGLs. These findings are in line with data from Amar et al. who demonstrated a recurrence rate of 6.5% at 5 years and 16.1% at 10 years [13]. Moreover, 17.9% of cases in our cohort experienced their recurrence after 15 years (three TAPGLs, one HNPGL, and three PHEOs). One patient was diagnosed with recurrence 48 years after the initial resection of a 6 cm right PHEO at the age of 17. She had a functional metastatic recurrence, with recurrence in the surgical bed and in multiple lymph nodes at the age of 65. Germline genetic analysis for *VHL*, *RET, SDHB, SDHC*, and *SDHD* genes did not reveal any mutations. Very late cases of recurrent PPGLs have been described, including a case by Thai et al. that described the late recurrence of an apparently benign PHEO removed surgically 25 years earlier [27]. Half of TAPGLs patients experienced their recurrence after 15 years. Johnston et al. reported a TAPGL that recurred 17.7 years after initial surgery [21]. This further supports a lifelong follow-up for patients who underwent operations for TAPGLs.

Younger age at diagnosis has been associated with a higher risk of recurrence [15]. In our study, this was not always observed. In fact, 8/20 (40%) recurrent PHEOs and 6/13 (46.2%) HNPGLs were over 50 years old at the initial diagnosis of PPGL. However, there were no cases of recurrent TAPGLs who were over 50 years old at the initial diagnosis. Tumor size of more than 5 cm has also been shown to be a predictor of recurrence [25]. In a study by Press et al., patients with PHEOs of over 5 cm had a recurrence rate of 11.5% compared to 0% in patients with PHEOs under 5 cm [25]. In our cohort, average PHEO size and TAPGL size were both above 5 cm (8.2 and 9.6 cm, respectively). However, three (17.6%) recurrent PHEOs had initial tumor sizes that were under 5 cm (3–3.5 cm) and among them, genetic status was known for one patient and showed no mutation. One TAPGL was under 5 cm at the initial diagnosis. This patient harbored a *SDHB* germline mutation. As expected for HNPGLs, tumors tended to be smaller with an average size of 2.7 cm. Only one of the thirteen recurrent HNPGLs was greater than 5 cm.

**Figure 5:**
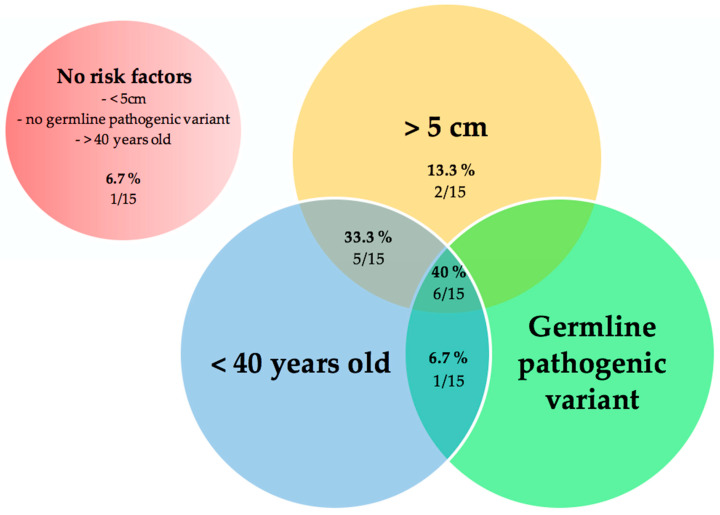
Venn diagram according to known risk factors for recurrent PHEOs/TAPGLs. Notes: recurrent PHEOs/TAPGLs for whom information for size, age at diagnosis, and genetic status was known (*n* = 15). PHEOs: pheochromocytomas, TAPGLs: thoracoabdominal paragangliomas.

In our cohort, we had 15 recurrent PHEOs or TAPGLs that had all the information regarding size, genetic status, and age at diagnosis. Forty percent of these patients (6/15) had all three risk factors (<40 yo, initial tumor > 5 cm and harbored a germline pathogenic variant) and 6.7% (1/15) had none. Eighty percent of patients had at least two risk factors and two patients had size as their only risk factor (Figure 5).

Overall, 46.2% of the recurrences were local and 53.8% were metastatic. PHEOs had metastatic recurrences in 75% of cases compared to 15.4% in HNPGLs and 66.7% in TAPGLs. A study by Timmers et al. showed that more than 75% of recurrent PHEOs were metastatic [23], and Contrera et al. showed that HNPGLs were more likely to have local recurrences [26].

Carrying a predisposing genetic mutation or syndromic presentation has also been linked to a higher risk of recurrence [15,17]. In our study, not all patients before 2014 underwent systematic genetic testing. This seems to be similar to other cohorts. For example, in the study by Parasiliti-Caprino et al., 46.3% of the cohort underwent genetic testing [15]. In our cohort of patients with recurrent PHEOs, the mutation rate was 36.4% (4/11). The most frequent mutation was identified in the *RET* gene (MEN2A) (three *RET* and one *FH*). In a cohort of 52 PHEOs associated with *MEN2A* mutations, the recurrence rate was 18.5%, which is higher than the general recurrence rate. Recurrent PGLs had a mutation rate of 66.7% (8/12) including four HNPGLs and four TAPGLs (three *SDHB*, two *SDHC*, two *SDHA*, and one *SDHD*). Not surprisingly, *SDHB* was the most frequent gene identified as mutated in accordance with previous studies where *SDHB* mutation was linked to a higher local and metastatic recurrence rate [28].

The main limitation of our study was its retrospective nature. While this allowed for a long-term follow-up of patients, it also limited the data available, such as initial biochemical status (including 3-methoxytyramine dosing) and genetic status. A total of 197 patients were followed at another center or lost to follow-up, further limiting access to some information and making the percentage of recurrences difficult to interpret. Moreover, as a quaternary center, we are subject to referral bias since we receive referred complex cases throughout the province, possibly raising our recurrence rate.

## 5. Conclusions

In conclusion, in our cohort of patients with recurrent PPGLs, a majority had recurrences after 5 years of follow-up, with a third of them recurring after 10 years. Germline mutation rates were higher in our recurrence cohort than in the general PPGL population. While tumor sizes over 5 cm are associated with an increased risk of recurrence, 4/22 (18.2%) of patients with PHEOs or TAPGLs in our cohort had a recurrence with an initial tumor measuring less than 5 cm. Moreover, the best modalities have yet to be determined since annual biochemical screening may not be sufficient as illustrated here with 2 PHEO cases in which imaging revealed recurrences before the elevation of catecholamines. Thus, a follow-up approach based on patient and tumor characteristics remains complex, as neither size, age nor mutational status are perfect predictors of recurrences. We believe that overall, the safest option remains a lifelong follow-up. However, patients and physicians should be involved in a shared follow-up decision based on risk factors and weighed against potential stressors or costs.

## Figures and Tables

**Table 1:** Genetic description of patients with and without recurrent PPGLs.

	Recurrent PPGLs Who Underwent Genetic Testing (*n* = 23) *		PPGLs With No Recurrences Who Underwent Genetic Testing (*n* = 66)	
Genes with Germline Pathogenic Variant, *n* (%)	12 (52.2)		21 (31.8)	
	*n* (%)	Delay to Recurrence in Months Mean (Min–Max)	*n* (%)	Follow-Up Time in Months Mean (Min–Max)
*NF1*, *n* (%)	0 (0.0)	-	5 (23.8)	131 (66–233)
*RET*, *n* (%)	3 (25.0)	113 (14–240)	4 (19.0)	208.5 (172–234)
*SDHB*, *n* (%)	3 (25.0)	143 (26–288)	4 (19.0)	51 (30–61)
*SDHC*, *n* (%)	2 (16.7)	91.5 (39–144)	3 (14.3)	157 (101–232)
*MAX*, *n* (%)	0 (0.0)	-	2 (9.5)	91.5 (31–152)
*FH*, *n* (%)	1 (8.3)	60	1 (4.8)	42
*SDHA*, *n* (%)	2 (16.7)	119 (58–180)	1 (4.8)	196
*VHL*, *n* (%)	0 (0.0)	-	1 (4.8)	146
*SDHD*, *n* (%)	1 (8.3)	38	0 (0.0)	-
No pathogenic variant, *n* (%)	10 (43.5)		38 (57.6)	
Variant of unknown significance, *n* (%)	0 (0)		7 (10.6)	

* One patient with a recurrent PPGL has pending genetic results.

**Table 2:** Characteristics of patients with recurrent PPGLs.

	PHEOs N = 20	HNPGLs N = 13	TAPGLs N = 6
Female n (%)	14 (70.0)	11 (84.6)	2 (33.3)
Age at diagnosis (y) Mean (min–max)	42.4 (16–67)	46.2 (27–57)	30.3 (15–45)
Age at recurrence (y) Mean (min–max)	53.25 (19–78)	53 (30–71)	45.2 (27–60)
Delay to recurrence (months) mean;median (min–max)	120.9; 77.5 (14–584)	82.4; 67 (24–180)	176.7; 188 (26–312)
Mean of maximal tumor diameter at initial diagnosis (cm) (min–max) *	8.2 (3–24)	2.7 (0.2–7.5)	9.6 (3–14)
Local recurrence *n* (%)	5 (25.0)	11 (84.6)	2 (33.3)
Metastatic recurrence *n* (%)	15 (75.0)	2 (15.4)	4 (66.7)

PHEO: Pheochromocytoma, HNPGL: head and neck paraganglioma, TAPGL: thoracoabdominal paraganglioma. * Tumor diameter: missing data for 3 patients for PHEOs, 1 patient for HNPGL, 1 TAPGL.

**Table 3:** Overview of recurrence rate for PHEOs and PGLs reported in the literature.

	Population	Recurrence Rate	Tumor Types	Predictors of Recurrence	Time of Recurrence	Local or Metastatic
Van Heerden et al., 1990 [22]	98 PPGLs	6.5%	6 PHEOs	-	5–13 years	33.3% local 66.6% metastatic
Amar et al., 2005 [13]	192 PPGLs	5 year: 6.5% 10 year: 16.1%	22 PHEOs 7 PGLs	Age Tumor site Familial disease	-	48.3% local 51.7% metastatic
Timmers et al., 2008 [23]	69 PHEOs	13.0%	9 PHEOs	-	1–14 years	22.2% local 77.8% metastatic
Shen et al., 2010 [24]	102 PPGLs	6.9%	7 PHEOs	-	6 months–17 years	100% local 0% metastatic
Press et al., 2014 [25]	135 PHEOs	6%	8 PHEOs	tumor size > 5 cm	7–106 months	25% local 75% metastatic
Johnston et al., 2015 [21]	52 PHEOs and TAPGLs	5.8%	2 PHEOs 1 TAPGL	-	8–17.7 years	100% local
Amar et al., 2016 [17] * Meta-Analysis	38 studies 2396 curative surgeries for PHEOs or TAPGLs. Median of 94% PHEOs.	1–34% (median 6%)	-	Syndromic presentation PGLs	Median 60 months	-
Contrera et al., 2020 [26]	189 HNPGLs	4 years: 8.2% 10 years: 17.1%	42 HNPGLs	Tumor site	Median 18.4 years	90.5% local 9.5% metastatic
Parasiliti-Caprino et al., 2020 [15]	242 PPGLs	17.4%	35 PHEOs 7 PGLs	Genetic mutation Large tumors	Median 2.9 years	59.5% local 40.5% metastatic
Holscher et al., 2020 [18] * Meta-analysis	13 studies 430 PPGLs	3%	-	-	Median 49.4 months	25% local 75% metastatic
Parisien-La Salle et al., 2022	309 PPGLs	12.6%	20 PHEOs 13 HNPGLs 6 TAPGLs	-	1–48 years	46.2% local 53.8% metastatic

## Data Availability

Some or all datasets generated during and/or analyzed during the current study are not publicly available but are available from the corresponding author on reasonable request.
